# Diagnosis of Breast Cancer Tissues Using 785 nm Miniature Raman Spectrometer and Pattern Regression

**DOI:** 10.3390/s17030627

**Published:** 2017-03-19

**Authors:** Qingbo Li, Can Hao, Zhi Xu

**Affiliations:** 1 School of Instrumentation Science and Opto-Electronics Engineering, Precision Opto-Mechatronics Technology Key Laboratory of Education Ministry, Beihang University, Xueyuan Road No. 37, Haidian District, Beijing 100191, China; 2 Department of General Surgery, Third Hospital, Peking University, Beijing 100083, China; zy1517312@buaa.edu.cn

**Keywords:** Raman spectrometer, breast cancer, pattern recognition, adaptive net analyte signal weight K-local hyperplane (ANWKH)

## Abstract

For achieving the development of a portable, low-cost and in vivo cancer diagnosis instrument, a laser 785 nm miniature Raman spectrometer was used to acquire the Raman spectra for breast cancer detection in this paper. However, because of the low spectral signal-to-noise ratio, it is difficult to achieve high discrimination accuracy by using the miniature Raman spectrometer. Therefore, a pattern recognition method of the adaptive net analyte signal (NAS) weight k-local hyperplane (ANWKH) is proposed to increase the classification accuracy. ANWKH is an extension and improvement of K-local hyperplane distance nearest-neighbor (HKNN), and combines the advantages of the adaptive weight k-local hyperplane (AWKH) and the net analyte signal (NAS). In this algorithm, NAS was first used to eliminate the influence caused by other non-target factors. Then, the distance between the test set samples and hyperplane was calculated with consideration of the feature weights. The HKNN only works well for small values of the nearest-neighbor. However, the accuracy decreases with increasing values of the nearest-neighbor. The method presented in this paper can resolve the basic shortcoming by using the feature weights. The original spectra are projected into the vertical subspace without the objective factors. NAS was employed to obtain the spectra without irrelevant information. NAS can improve the classification accuracy, sensitivity, and specificity of breast cancer early diagnosis. Experimental results of Raman spectra detection in vitro of breast tissues showed that the proposed algorithm can obtain high classification accuracy, sensitivity, and specificity. This paper demonstrates that the ANWKH algorithm is feasible for early clinical diagnosis of breast cancer in the future.

## 1. Introduction

Approximately 231,840 new cases of invasive breast cancer and 40,290 breast cancer deaths were estimated to occur among US women in 2015, according to the data provided by the American Cancer Society. Aside from skin cancers, breast cancer is the most common cancer diagnosed among US women, accounting for nearly one in three cancer cases. Breast cancer is also the second leading cause of cancer death among women, after lung cancer [1]. In 2015, approximately 260,000 new cases of breast cancer were diagnosed and 70,000 breast cancer deaths occurred in China. This incidence ranked first in the female malignant tumors [2]. The data above show that early breast cancer diagnosis is important in implementing timely, effective, and early medical treatments.

In the operation, the traditional pathological diagnostic method is the frozen section pathological diagnosis. However, pathological diagnosis requires waiting a long time and cannot meet the requirements of rapid diagnosis. In addition, the tumor margin cannot be accurately and quickly ascertained, which makes the recurrence rate high and patients need a second operation in a short time. As a form of molecular spectroscopy, Raman spectroscopy can detect cancer-induced changes in the molecular structure and composition of breast tissue. Raman spectroscopy also exhibits characteristics such as sharp peaks, a lack of interference of water, a low amount of samples required, and no chemical treatment of samples required [3,4,5,6,7]. Raman spectroscopy has potential for the early and rapid clinical diagnosis of breast cancers at the molecular level because some chemical changes (e.g., in carbohydrates, lipids, proteins, and nucleic acids) occur during tumor formation.

As a diagnostic technology for breast cancers, Raman spectroscopy has been developed recently. Many investigations on Fourier transform Raman spectroscopy (FTRS), confocal Raman microspectroscopy (CRS), resonance Raman spectroscopy (RRS), surface-enhanced Raman spectroscopy (SERS), and conventional Raman spectroscopy for breast cancer diagnosis have been performed during the development of diagnostic technology [8,9,10,11,12,13,14,15]. SERS, FTRS, CRS, and RRS have been extensively investigated as breast cancer diagnosis tools for allowing reduced fluorescence and high resolution of the Raman spectra [8,9,10,11,16,17,18]. However, due to requiring a large Raman spectrometer or a large desktop microscope, these technologies increase the costs and lessen the portability of clinical diagnosis. Combined with an optical fiber probe, conventional Raman spectrometers are small in size, portable, and low in cost, and hold much promise for in vivo and in situ cancer detection. However, the Raman spectra obtained by the miniature Raman spectrometers exhibit a strong fluorescence background interference and a low spectral signal-to-noise ratio. Both increase the difficulty of data processing and decrease the discrimination accuracy of common data analysis methods. Therefore, considering these key problems for detecting breast cancer, investigating the discrimination analysis method for high classification accuracy is significant.

In this paper, a novel algorithm of adaptive net analyte signal weight K-local hyperplane (ANWKH) is proposed to discriminate cancerous and normal human breast tissue Raman spectra. ANWKH is an extension and improvement of K-local hyperplane distance nearest-neighbor (HKNN) [19]. Although HKNN performs well only for small values of K, it suffers from bias for data with high dimensions [20]. Although AWKH is the improvement of the HKNN method, there is noise, fluorescence, and other mixed component interference. These factors will not only increase the calculation, but also lower the calculation accuracy. It is necessary to improve the AWKH method. ANWKH combines the advantages of the adaptive weight k-local hyperplane (AWKH) and the net analyte signal (NAS) [21]. In this algorithm, NAS was first used to eliminate the interference caused by other non-target factors. The key factors are the biomolecules such as lipids, proteins, and nucleic acids. Other non-target factors are noise interference, fluorescence background, and other components of tissue. Then, the distance between the test set samples and hyperplane was calculated with consideration of the feature weights. The feature weight is estimated by using the ratio of the between-group to with-group sums of squares for the data assigned to the given classes. The HKNN works well only for small values of the nearest-neighbor. However, the accuracy decreases with increasing values of the nearest-neighbor. The method presented in this paper can resolve the basic shortcoming through using the feature weights. The original spectra are projected into the vertical subspace without the target factors. NAS was employed to obtain the spectra without irrelevant information. NAS can improve the classification accuracy, sensitivity, and specificity of the early diagnosis of breast cancer. Consequently, ANWKH is effective for discriminating cancerous and normal human breast tissue Raman spectra.

## 2. Materials and Methods

### 2.1. Tissue Specimens

Normal and cancerous samples of human breast tissue were obtained from female patients in Peking University Third Hospital, including four normal tissues and twelve cancerous tissues. The tissues were sourced from female patients aged from 33 years old to 88 years old (mean age is 56 years old). The samples were preserved in liquid nitrogen after the spectra were acquired and sent for the pathological diagnosis as the reference in the spectral analysis. These spectra were supported by pathological analysis (hematoxylin-eosin stain) results.

### 2.2. Raman Spectral Measurements

The conventional Raman spectra were acquired by an Ocean Optics QE65Pro miniature fiber optic Raman spectrometer at a 785 nm excitation wavelength. Without any chemical treatment, specimens were frozen using liquid nitrogen. They were placed in the glass slide for Raman spectral measurement after thawing at room temperature. The thickness of the sample is about 2 cm. The penetration depth of the probe is about the micron level. The laser power used for acquisition was 30 mW. The integration time was 30 s. All spectra were acquired at a wavelength range from 700 cm^−1^ to 2000 cm^−1^. 700–2000 cm^−1^ is known as the fingerprint region, which contains complete information about the biomolecules such as lipid, protein, nucleic acids, etc. For objective data, every sample was measured at different locations. Three spectra were also measured at every same location and then averaged to reduce the noise level. In total, 131 sample spectra were obtained from four normal tissues and twelve cancerous tissues on the same environmental conditions on two days (a total of 139 spectra, except for eight outlier spectra samples acquired improperly with other laser powers, which were made by human behaviors’ error.). Up to 73 Raman spectra (16 normal and 57 cancerous) were obtained in the first day, and 58 Raman spectra (18 normal and 40 cancerous) were obtained in the second day.

### 2.3. Spectra Preprocessing Method

Noisy and fluorescence background occurs in the spectra collected by the Ocean Optics QE65Pro Raman spectrometer. Thus, the spectra were preprocessed before use. First, the noise was removed by wavelet transform; then, the fluorescence background was removed by fitting the smoothed spectra to a third-order polynomial function; third, the data sets were normalized to zero mean and unit variance. Data preprocessing can enable clear spectral peaks and optimize the spectral quality. 

### 2.4. Discrimination Analysis Method

In the paper, ANWKH algorithm is proposed. ANWKH algorithm is an improvement and extension of HKNN algorithm. As HKNN performs badly for data with high dimensions, ANWKH resolves the problem and obtains high accuracy by combining the feature of AWKH and NAS. Before the use of the AWKH algorithm, NAS was first employed to eliminate constituents caused by other non-target parameters, such as the noise, background interference, and other components interference. The original spectra are projected into the subspace with various interference factors but without the objective factors to obtain the spectra without irrelevant information. Then, the Euclidean distances between the test set samples and hyperplane were calculated after considering the feature weights estimated by using the ratio of the between-group to with-group sums of squares. The nearest neighbors are selected by the weighted Euclidean distance between the test sample and training set. Finally, the class labels are distinguished according to the Euclidean distances between the test set samples and hyperplane.

The ANWKH algorithm specific procedures are as follows:

Suppose that the training set X consists of L samples with J classes. Each training sample consists of d input features $X_{i}={\left(X_{i1},...,X_{id}\right)}^{T}$ with known class label $y_{i}=c(i=1,...,L;c=1,...,J)$. The goal is to predict the class label of a query with input vector $q={\left(q_{1},...,q_{d}\right)}^{T}$.

Step 1 The original spectra are reconstructed based on the first f principal components. Then, they are projected into the subspace $X_{-k}$ with various interference factors but without the objective factors. The net spectra x according to the following formulas were then calculated [22]. Each training sample consists of d input features $x_{i}={\left(x_{i1},...,x_{id}\right)}^{T}$ with known class label $y_{i}=c(i=1,...,L;c=1,...,J)$.
  (1) $$\begin{array}{l} x=X[I-{\left(X_{-k}^{+}\right)}^{T}\times X_{-k}]\\ X_{-k}=X(I-\beta \times X^{+}\times y\times \bar{X})\\ \beta =\frac{1}{\bar{X}\times X^{+}\times y} \end{array}$$
  where I is an identity matrix and $X_{-k}^{+}$ is the generalized inverse matrix of $X_{-k}$, which is the subspace with various interference factors but without the objective factors. Besides, y denotes the training sample class label, $\bar{X}$ denotes the mean spectrum of the training sample set, and β is a scalar.
Step 2 Calculating the feature weight w of the training sample, the formulas are as follows [23,24]:
  (2) $$\begin{array}{l} r_{j}=\frac{\sum_{i}\sum_{c}I(y_{i}=c){\left({\bar{x}}_{cj}-{\bar{x}}_{j}\right)}^{2}}{\sum_{i}\sum_{c}I(y_{i}=c){\left(x_{ij}-{\bar{x}}_{cj}\right)}^{2}}\\ w_{j}=\frac{\exp(r_{j})}{\sum_{j=1}^{d}\exp(r_{j})}\;\forall j=1,...,d \end{array}$$
  where ${\bar{x}}_{j}$ denotes the jth component of the grand class centroid and ${\bar{x}}_{cj}$ denotes the jth component of class centroid of class c. I(·) denotes the indicator function equals to 1 when $y_{i}=c$. Otherwise, it is equal to 0. $x_{ij}$ denotes the jth component of the ith training sample.
Step 3 Calculating the weighted Euclidean distance metric D between training samples and the test samples according to the following formula:
  (3) $$D(x_{i},q)=\sqrt{\sum_{j=1}^{d}w_{j}{\left(x_{ij}-q_{j}\right)}^{2}}$$
Step 4 In accordance with the Euclidean distance D, we select K nearest neighbors of class c $p_{c}=(p_{c1},...,p_{cn_{c}})$ for the given query q. Then, we construct the local hyperplane of class c with $p_{c}$ as follows:
  (4) $$\begin{array}{l} LH_{c}(q)=\{s|s=\sum_{i=1}^{n_{c}}\alpha_{i}V_{\cdot j}+m_{c}\}\\ m_{c}=\frac{1}{n_{c}}\sum_{i=1}^{n_{c}}p_{ci}\\ V_{\cdot i}=p_{ci}-m_{c}\\ \alpha ={\left(\alpha_{1},...,\alpha_{n_{c}}\right)}^{T} \end{array}$$
Step 5 Calculating the minimum distance between q and $LH_{c}(q)$ according to the following formulas:
  (5) $$\begin{array}{ll} J_{c}(q) & =\underset{\alpha }{\min}\sum_{j=1}^{d}w_{j}{\left(V_{j\cdot }\alpha +m_{cj}-q_{j}\right)}^{2}+\lambda \alpha^{T}\alpha \\ & =\underset{\alpha }{\min}{\left(s-q\right)}^{T}W(s-q)+\lambda \alpha^{T}\alpha \\ W= & diag(w_{1},...,w_{d}) \end{array}$$
  where the regularization parameter λ is selected to avoid α being too large. We solve the equation $\frac{\partial J_{c}(q)}{\partial \alpha }=0$, then achieve the value α under the minimum distance by $\alpha =(U^{T}V+\lambda I_{n_{c}})/(U^{T}(q-m_{c}))$, where $U^{T}=V^{T}W$.
Step 6 Evaluating a class label to q by the formula as follows:
  (6) $$\mathrm{label}(q{)=\mathrm{argmin}}_{c}J_{c}(q)$$

## 3. Results and Discussion

### 3.1. Spectral Preprocessing

**Figure 1:**
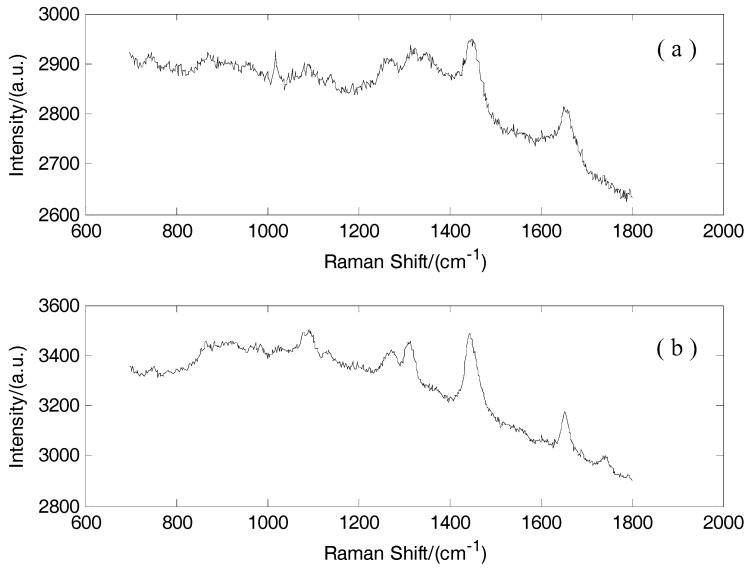
(**a**) Original Raman spectra of breast cancerous tissue without preprocessing; (**b**) Original Raman spectra of breast normal tissue without preprocessing.

**Figure 2:**
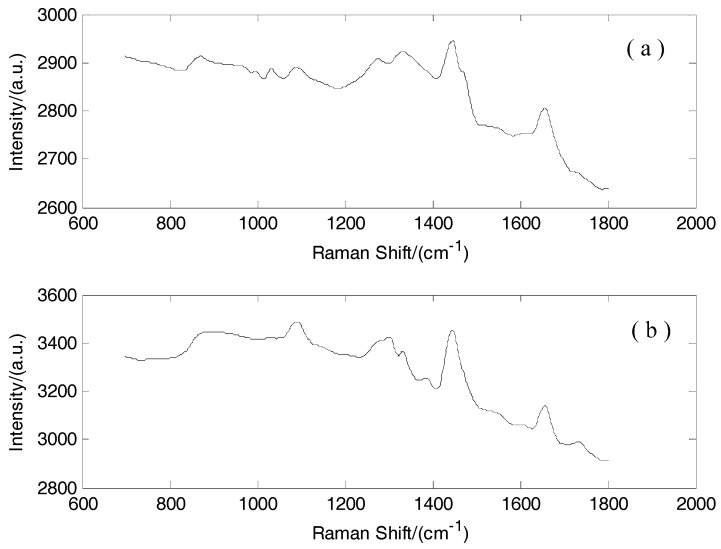
(**a**) Raman spectrum of cancerous tissue after wavelet transforms; (**b**) Raman spectrum of normal tissue after wavelet transforms.

**Figure 3:**
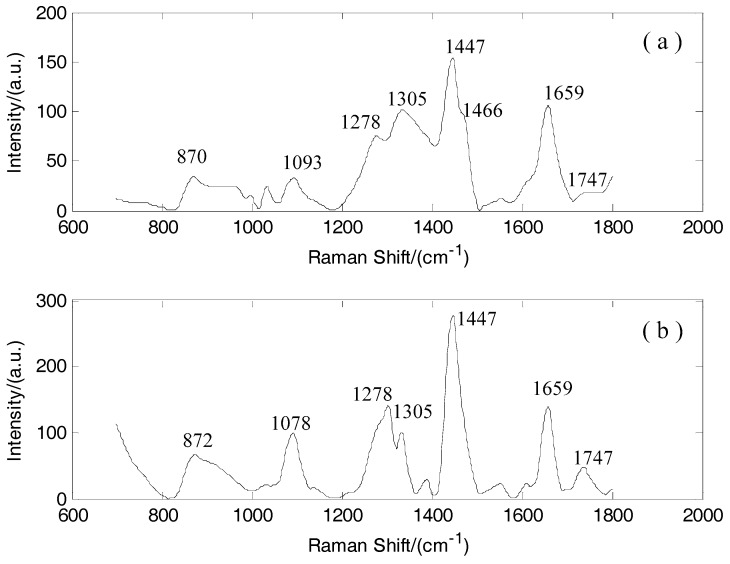
(**a**) Raman spectrum of breast cancerous tissues after preprocessing; (**b**) Raman spectrum of breast normal tissues after preprocessing.

From the original spectra without preprocessing (Figure 1), the noises and fluorescence backgrounds exhibited a serious influence on the spectra and decreased the discrimination accuracy. Thus, the noise was removed by wavelet transform. The Symmlet-5 wavelet filter and four-decomposition scales were adopted to smooth the Raman spectra (Figure 2). The fluorescence background was removed by a third-order polynomial function. The Raman spectra of normal and cancerous tissues after preprocessing are shown in Figure 3. The Standard Deviation values are also shown in Section 3.2. The normalization was performed across the samples. In order to eliminate the effect of different excitation light energy and spectral collection efficiency on the whole spectrum, it is convenient to compare the intensity of the spectra and different Raman signals.

The quality of the optimized Raman spectra was improved greatly after data preprocessing (Figure 1 and Figure 3). The Raman peaks of normal and cancerous tissues are pronounced after preprocessing. Thus, preprocessing can improve the discrimination accuracy.

The Raman peaks of normal tissues and cancerous tissues are shown in Figure 3. The Raman peaks of normal tissue occur at 872, 1078, 1305, 1447, 1659 and 1747 cm^−1^. Meanwhile, the Raman peaks of cancerous tissue occur at 870, 1093, 1278, 1305, 1447, 1466, 1659 and 1747 cm^−1^. 

According to the research made by using the Raman spectroscopy to measure normal breast tissues and cancerous breast tissues, normal tissue spectra are attributable to lipid molecules, whereas cancerous tissue spectra are attributable to protein molecules. For example, the peak at 1663 cm^−1^ represents amide I, one of the protein molecules. The peak at 1278 cm^−1^ indicates the presence of amide III(C-N stretch), and the peak at 1453 cm^−1^ indicates the presence of CH_2_ deformation. The main differences between normal breast tissue spectra and cancerous breast tissue spectra are shown. The peak position representing protein molecules appears at 1278 cm^−1^ in cancerous tissues and nearly disappears in normal tissues. Besides, normal tissue shows a sole prominent lipid peak at 1447 cm^−1^ and 1659 cm^−1^, where the peak intensities in cancerous tissues decrease obviously compared with those in normal tissues. Changes have occurred in the configurations, components, and quantities of proteins, lipids, and nucleic acids during tumor formation [25,26]. Normal tissues contain more lipids, whereas cancerous tissues contain more relative proteins. This condition is the basis of breast cancer diagnosis.

Specific assignments of individual peaks can be found in Table 1.

### 3.2. Statistical Analysis

The data after preprocessing were separated into two parts. One was the training set and the other was the test set. The STD Dev value of all breast cancerous spectra was 0.0232, and the STD Dev value of all breast normal spectra was 0.1797. Each classifier was learned on the training set and applied on the test set.

The entire spectra were preprocessed at first. Then, the 73 Raman spectra (16 normal and 57 cancerous) obtained on the first day were selected as the training set, and the 58 Raman spectra (18 normal and 40 cancerous) obtained on the second day were selected as the test set. Third, the training and test sets were normalized to zero mean and unit variance. Finally, the test set was classified by ANWKH, AWKH, HKNN, and support vector machine (SVM) classifiers, respectively.

The daily classification results are shown in Table 2.

The entire data were preprocessed initially and normalized to zero mean and unit variance. Data were then classified by ANWKH, AWKH, HKNN, and SVM classifiers, respectively. Finally, the cross-verification method was used to verify the discrimination accuracy. The results are shown in Table 3.

Data processing was conducted two more times. The total 131 Raman spectra were split into two data sets randomly 10 times. Every time, 87 Raman spectra after preprocessing were selected as the training set. The other 44 Raman spectra after preprocessing were selected as the test set. Subsequently, the algorithms were examined. Table 4 shows the average accuracy of the 10 experiments using four different methods with optimal parameters.

As shown in Table 2 and Table 3, ANWKH achieved the highest accuracy among the four different classifiers, and AWKH came second. In the experiment, the discrimination accuracy by ANWKH was 93.1%, the sensitivity was 99.2%, the specificity was 79.7%, the positive predictive value was 91.6%, and the negative predictive value was 97.9%. The average results of the random classification for ANWKH, AWKH, HKNN, and SVM are shown in Table 4. It shows that ANWKH values were the highest between the four classifiers on accuracy, sensitivity, specificity, the positive predictive value, and the negative predictive value. The results by ANWKH were much more accurate than the ones by AWKH.

From the experimental results above, ANWKH shows a great advantage for classifying the Raman spectra of breast tissues. The spectra with irrelevant or redundant features can be classified accurately with ANWKH because it eliminates the influence caused by other non-target factors and considers the feature weights. SVM can perform well with large-scale data. However, the choices of the parameters for the kernel are complex and unstable. The HKNN works well only for small values of the nearest-neighbor, but the accuracy decreases with the values of the increasing nearest-neighbor. AWKH can improve the classification accuracy by using the feature weights, which is the improvement of the HKNN method. However, there are noises, fluorescence, and other mixed component interference. These factors will not only increase the calculation, but also lower the calculation accuracy. Consequently, ANWKH is effective and worth studying.

## 4. Conclusions

Raman spectroscopy, as a sensitive probe on the molecular level, can achieve the early diagnosis of breast cancer and ascertain the tumor margin accurately and quickly at the early stage of tumor formation. The miniature laser Raman spectrometer with a 785 nm excitation is easily used in the clinical diagnosis of breast cancer because of its advantages, such as small size, portability, and low cost. Some disadvantages are strong fluorescence background interference and a low spectral signal-to-noise ratio, which increase the difficulty of data processing and decrease the discrimination accuracy of the data analysis methods. Thus, it is important to investigate the discrimination analysis method for high classification accuracy.

The fundamental experiment results show that the proposed classification algorithm is effective. First, the conventional Raman spectra of breast tissues were acquired by the miniature laser Raman spectrometer at a 785 nm excitation. Then, the preprocessing procedures were investigated. Finally, a novel classification algorithm, ANWKH, was proposed. In this algorithm, NAS was employed to obtain the spectra without irrelevant information. The original spectra are projected into the vertical subspace without the target factors, which can eliminate the irrelevant information caused by other non-target parameters. Then, the distance between the test set samples and hyperplane was calculated after considering the feature weights. The HKNN works well only for small values of the nearest-neighbor. However, the accuracy decreases with the increasing values of the nearest-neighbor. The method presented in this paper can resolve the basic shortcoming through using the feature weights. The experimental results in vitro indicate that ANWKH achieved high classification accuracy, although the Raman spectra obtained by the miniature laser Raman spectrometer exhibited strong fluorescence background interference and a low spectral signal-to- noise ratio. It is proved that it is viable, rapid and accurate for breast cancer diagnosis in vivo and in situ with a miniature laser Raman spectrometer. In the future, the miniature spectrometer can be used for breast cancer diagnosis in vivo and in situ and can ascertain the tumor margin to relieve female breast cancer patients’ pain.

## Figures and Tables

**Table 1:** Peak positions and assignments of breast tissue.

Peak Position (cm^−1^)	Major Assignment
1078	C–C or C–O stretch (lipid)
1278	Amide III(C–N stretch) (protein)
1305	Amide III, α-helix, C–C str and C–H (protein)
1447	Scissoring mode of methylene (CH_2_) (lipid)
1453	CH_2_ deformation (protein)
1653	lipid
1663	Amide I(C=O stretch) (protein)
1747/1750	C=O stretch (lipid)

**Table 2:** The daily classification results of the test set for ANWKH, AWKH, HKNN, and SVM.

Method	Sensitivity (%)	Specificity (%)	The Positive Predictive Value (%)	The Negative Predictive Value (%)	Accuracy (%)
SVM	92.5	61.1	84.1	78.5	82.75
HKNN	90.0	66.7	85.7	75.0	82.76
AWKH	95.0	72.2	88.4	86.7	87.93
ANWKH	99.2	79.7	91.6	97.9	93.10

**Table 3:** Cross-verification classification results of the test set for ANWKH, AWKH, HKNN, and SVM.

Method	Sensitivity (%)	Specificity (%)	The Positive Predictive Value (%)	The Negative Predictive Value (%)	Accuracy (%)
SVM	96.9	85.3	94.95	90.6	93.89
KNN	97.9	97.1	99.0	94.3	97.71
AWKH	99.0	97.1	99.0	97.1	98.47
ANWKH	99.0	100	100	97.1	99.24

**Table 4:** The average results of random classification for ANWKH, AWKH, HKNN, and SVM.

Method	Sensitivity (%)	Specificity (%)	The Positive Predictive Value (%)	The Negative Predictive Value (%)	Accuracy (%)
SVM	95.1	71.9	90.4	82.6	92.53
KNN	95.9	68.0	89.6	85.0	88.63
AWKH	95.0	74.0	92.2	82.0	93.18
ANWKH	97.1	82.4	94.1	89.8	94.83
